# New Fiber Bragg Grating Three-Dimensional Accelerometer Based on Composite Flexure Hinges

**DOI:** 10.3390/s21144715

**Published:** 2021-07-09

**Authors:** Hui Wang, Lei Liang, Xiongbing Zhou, Bin Tu

**Affiliations:** 1 National Engineering Laboratory for Fiber Optic Sensing Technology, Wuhan University of Technology, Wuhan 430070, China; wanghui1989@whut.edu.cn (H.W.); zhouxiongbing@whut.edu.cn (X.Z.); 2 Advanced Engineering Technology Research Institute, Wuhan University of Technology, Zhongshan 528437, China; 291875@whut.edu.cn

**Keywords:** three-dimensional accelerometer, fiber bragg grating, flexure hinges, dynamic response

## Abstract

Multi-dimensional acceleration sensors are used in important applications in the aerospace, weapon equipment, and nuclear fields and have strict requirements in terms of performance, volume, and mass. Fiber Bragg grating acceleration sensors use optical wavelength signals as a medium for information transmission to effectively eliminate the influence of electromagnetic interference between multi-dimensional sensors. In this study, we designed a composite flexure hinge three-dimensional acceleration sensor. To this end, we investigated the coupling mechanism between a new integrated elastomer structure and fiber grating to determine the influence of structural parameters on the static and dynamic characteristics, volume, and mass of the sensor. By optimizing the strain distribution, amplitude, and frequency and coupling characteristics between dynamic dimensions, a design theory and a method for integrating the three-dimensional acceleration sensor were developed. The size of the optimized accelerometer is only 25 mm × 25 mm × 30 mm. Performance testing revealed that, along the three spatial dimensions, the sensor had sensitivities of 51.9, 39.5, and 20.3 pm/g, respectively, resonance frequencies of 800, 1125, and 1750 Hz, respectively, and a measurable frequency range of 0–250 Hz.

## 1. Introduction

In recent years, multi-dimensional accelerometers have been increasingly used for motion detection and control in undefined environments. Such accelerometers are being used in the structural health monitoring of aircrafts, missile weapons, building bridges, large industrial equipment, and other key equipment and facilities [1].

Multidimensional fiber bragg grating (FBG) acceleration sensors are aggregations of single-dimensional sensors and can be categorized in terms of structural form into combined- or integrated-type sensors [2]. Morikawa et al. [3] designed a three-axis FBG accelerometer in which a mass block is placed in the center of the sensor and the FBG is fixed in six directions. When the sensor is excited, the strains on the FBG along these six directions change to enable the measurement of three-dimensional acceleration at a frequency of 800 Hz while eliminating the influence of temperature on the sensitivity of measurement. Zhao et al. [4] theoretically analyzed a mathematical model of Morikawa’s fiber grating-based method of three-dimensional acceleration measurement and proved that neither the lateral effect nor the temperature of the sensor head has a significant effect on the measurement results and can therefore be ignored. Tests by Ai et al. [5] revealed that the sensitivity of a Morikawa-type three-dimensional acceleration sensor is 3.2 pm/g in each direction and the measurable frequency range is approximately 0–840 Hz. Feng et al. [6] added a metal bellows to this structure to connect the mass block and metal shell and avoid optical fiber fracturing as a result of excessive movement of the mass block. Guo et al. [7] designed a two-dimensional FBG acceleration sensor based on a steel-pipe mass-block moving structure. In their design, four FBGs are pasted onto a steel pipe along its axial direction and the FBGs located at two symmetrical positions are used to measure the change in acceleration along one axial direction. The sensor axial in this configuration is 8.6 pm/g and the acceleration measurement range is 1.5–7.6 g. Antunes et al. [8] modified the single-axis hinged acceleration sensor design by adding a multi-axis hinge to obtain a two-dimensional FBG acceleration sensor. The two axial FBGs used in this design have resonance frequencies of 845.33 and 846.01 Hz, respectively, and sensitivities of approximately 87.848 and 92.351 pm/g, respectively. To increase the sensitivity of the multi-axis hinged two-dimensional acceleration sensor design, Wei et al. [9,10] arranged two arc-shaped multi-axis hinges at symmetrical positions on both sides of a sensor base. In this design, the four FBGs are no longer connected to the base, but are instead fixed to the mass blocks of the two multi-axis hinges. The sensitivity of this sensor design is 220 pm/g and the natural frequency is 814.3 Hz, with an interference between the two axes of only 4%. Umesh et al. [11] developed a design in which two cantilevered FBG accelerometers are fixed to a large mass block. When multi-dimensional vibrations occur, the two mass blocks vibrate separately to produce acceleration measurements in two dimensions. Wan et al. [12] designed a two-dimensional FBG accelerometer based on a design featuring a screw and two mass rings; the positions of the mass rings on the screw can be adjusted, allowing for sensitivities in the x- and y-directions of 16.1–152.3 and 16.4–150.7 pm/g, respectively, in the 40–100 Hz frequency range. Wang et al. [13] developed a bidirectional Bragg grating acceleration sensor design with a circular flexure hinge. The sensor has a resonant frequency and sensitivity of approximately 368 Hz and 107.3 pm/g, respectively, and a lateral anti-interference degree of 4.8%; the errors between the theoretical and actual values of resonant frequency and sensitivity are 4.2 and 7.0%, respectively. Cui et al. [14] proposed a direction-sensitive two-dimensional accelerometer based on multi-core fiber gratings. By monitoring the wavelength shift of three of the seven nuclei, including the central nucleus and two outer nuclei, which are not aligned, the vibration direction and acceleration information can be obtained simultaneously. Song et al. [15] proposed a two-dimensional FBG vibration sensor based on an orthogonal flexure hinge structure. The elastic element of the sensor comprises an inertial body, a flexure hinge pair, and a base, and adopts a small-sized design and wire cutting processing in which a 3-mm-long hinge structure is used to obtain higher working frequencies with reduced cross interference. The resonant frequencies of the sensor in the x- and y-directions are approximately 1275 and 1482 Hz, respectively, and the third-order natural frequency is 4932 Hz, which indicates that the sensor has high torsional stiffness and strong torsional vibration resistance. Guo et al. [16] have designed and developed a compact optical fiber accelerometer based on 3D printing technology. The sensitivity is 421.4 pm/g and the bandwidth is from 10 to 210 Hz. Liu et al. [17] presented a three-component (3C) low-reflection fiber Bragg grating accelerometer for CMN self-suppression. The common mode noise suppression effect of the accelerometer on three axes is 4.5 db higher than that of the traditional method, and the push–pull structure can double the effective signal amplitude and improve the signal-to-noise ratio. Zhou et al. [18] designed and manufactured a three-dimensional fiber vector accelerometer for vibration measurement. The fiber was etched with multi-core fiber by a femtosecond laser and phase mask. The working frequency band of the accelerometer is 10–220 Hz, and the maximum sensitivity is 355 pm/g. Chen et al. [19] proposed and demonstrated a novel two-dimensional reflective accelerometer based on standard single-mode fiber (SMF) and orthogonal cladding Bragg grating (FBG). The cladding FBG was written point by point by afemtoseconda laser point and runs parallel to the core. Li et al. [20] found that the resonance frequency of the fiber Bragg grating could be increased without reducing the sensitivity by shortening the fixed distance between the two ends of the fiber Bragg grating. The sensitivity is 1100 pm/g and the resonance frequency is 42 Hz. Wu et al. [21] proposed an isosceles triangle cantilever mass acceleration sensor with a high natural frequency and high sensitivity. The natural frequency is 8193 Hz and the sensitivity is 0.46 pm/g. Hong et al. [22] developed an M-type double cantilever structure which can produce a chirp effect and the sensor is insensitive to temperature. The sensitivity of the sensor is 256 pm/g and the natural frequency is 66 Hz. Yan et al. [23] proposed a novel fiber Bragg grating (FBG) accelerometer comprising two straight circular flexure hinges connected in parallel. The measurement range can reach 30–200 Hz and the sensitivity is 54 pm/g. Li et al. [24] summarized the research and development progress of fiber Bragg grating acceleration sensors and gave the performance index of single-dimensional, two-dimensional, and three-dimensional accelerometers.

Standard combined multi-dimensional sensors have strong lateral anti-interference abilities and operate following a simple principle. However, the scattered motion centers found in such sensors make the measurement of single-point acceleration inaccurate. In addition, such sensors occupy significant volume and are heavy and difficult to use. Although integrated FBG three-dimensional acceleration sensors are smaller and less heavy than standard designs, they feature large degrees of interference along each dimension. According to the latest report, the smallest size of a single-dimensional FBG acceleration sensor is 27 × 11 × 22 mm [10], with a sensitivity of 12 pm/g at 0–80 Hz frequency. Another one is 24 × 12 × 12 mm [25], with a sensitivity of 244 pm/g at the 0–40 Hz frequency range. The smallest size of a two-dimensional FBG acceleration sensor has encapsulated 24 × 24 × 24 mm [26], and the sensitivity is 9.4 pm/g in the 30–300 Hz frequency range. The other size that is a not fully encapsulated sensor is 45.5 mm × 125 um [14], and the sensitivity is 115.5 pm/g and 50.8 pm/g at 40 Hz frequency, respectively, while the smallest size of a 3D FBG acceleration sensor is a 130 × 28 mm cylindrical integrated structure [18].

In this study, the monitoring requirements and sensor weight and volume miniaturization requirements of the aerospace field were considered in the design of a hinged FBG three-dimensional acceleration sensor with an integrated structure. A small size usually means that the accelerometer performance will be influenced, thus proper accelerometer structures are needed to keep good performance. The hinge structure of this design is used to carry out the motion of the mass block. Following optimization of the sensor structural parameters and manufacturing process, the sensor was tested to determine the feasibility of practical application. The proposed sensor has the advantages of reduced volume and weight and low interdimensional interference.

## 2. Materials and Methods

### 2.1. Structural Design and Theoretical Derivation

**Figure 1:**
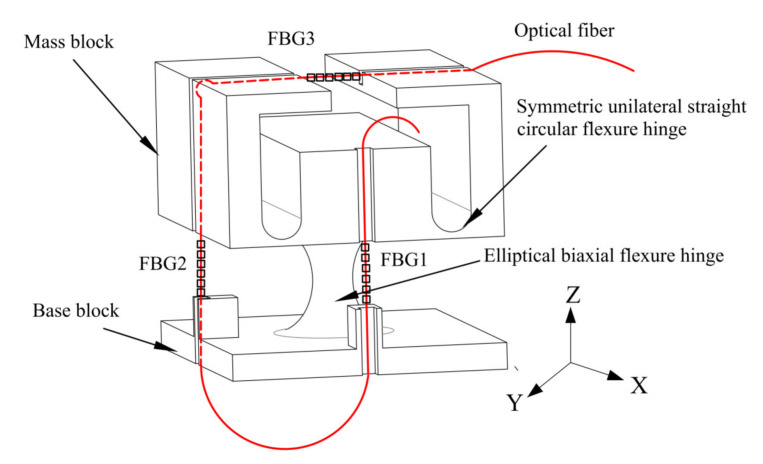
Three-dimensional accelerometer oscillator structure.

In the design of a multi-dimensional FBG acceleration sensor, it is necessary to ensure that the structure within the sensor can accommodate movement by each multi-dimensional sensor along all axes with respect to the measured body. To ensure measurement accuracy, the inter-directional degrees of mutual interference of the measurement mechanism should be as small as possible. The structure of the proposed FBG three-dimensional acceleration sensor is shown in Figure 1. The elastomer hinge structure comprises an elliptical biaxial flexure hinge and a symmetric unilateral straight circular flexure hinge.

The lower section of the sensor elastomer is a biaxial elliptical hinge structure in which two longitudinal fiber gratings are encapsulated on the two adjacent sides as acceleration measurement units in the x- and y-directions, respectively. Two completely symmetrical unilateral hinge structures are located above the elastomer, and a transverse fiber grating is encapsulated above the hinge to form the z-directional acceleration measurement unit. To ensure sensor measurement accuracy, the hinge and each side of the mass block are symmetrical around the center of the structure. In this section, we provide detailed theoretical derivations of the acceleration sensitivity and natural frequency of the three-dimensional accelerometer along each measurement direction to provide a theoretical basis for the structural parameter settings of the accelerometer.

FBGs are sensitive to environmental temperature change and strain (ε). At a constant environmental temperature, the shift in the center wavelength of the FBG can be expressed [27] as

(1) $$\Delta \lambda =\lambda_{B}\left(1-P_{e}\right)\varepsilon$$

where $\lambda_{B}$ is the Bragg wavelength and *P_e_* is the elasto-optical coefficient of the optical fiber.

#### 2.1.1. X- and Y-Directional Theoretical Formulas

The sensor base and mass block are located at the fixed and freely moving ends, respectively, of the elliptical double-axis flexible hinge sensing structure. The base and mass block are connected by the elliptical double-axis hinge; the FBG is enclosed within a space formed in the base of the double-axis hinge structure and a concave indentation in the mass block. When subjected to acceleration along the x-axis, the mass block rotates around the center of the y-axis of the hinge; when the mass block is subjected to acceleration along the y-axis, it rotates around the center of the hinge x-axis. The displacement induced by the motion of the mass block causes the fiber grating, which is sealed on both sides, to produce stress and strain that alters the FBG central wavelength.

**Figure 2:**
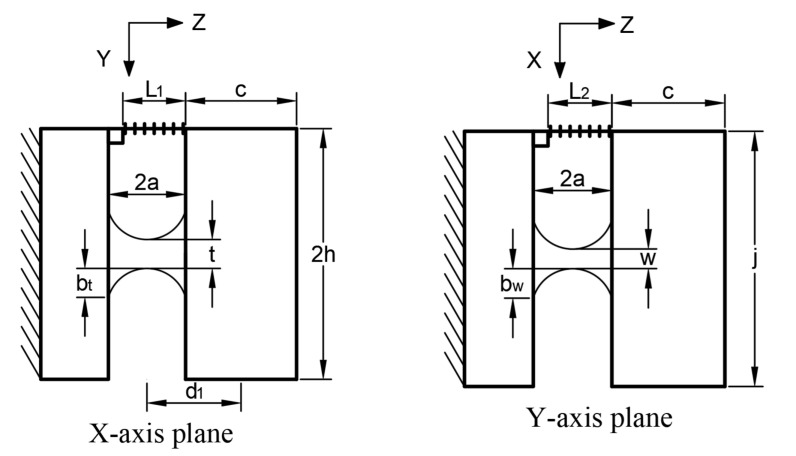
Simplified schematic of x-, y-directional sensor.

The influence of the groove encapsulating the fiber within the sensor on the elastomer can be ignored and the internal damping of the sensor can be assumed to be zero. The structural dimensions of the sensor’s elliptical biaxial hinge are shown in Figure 2.

**Figure 3:**
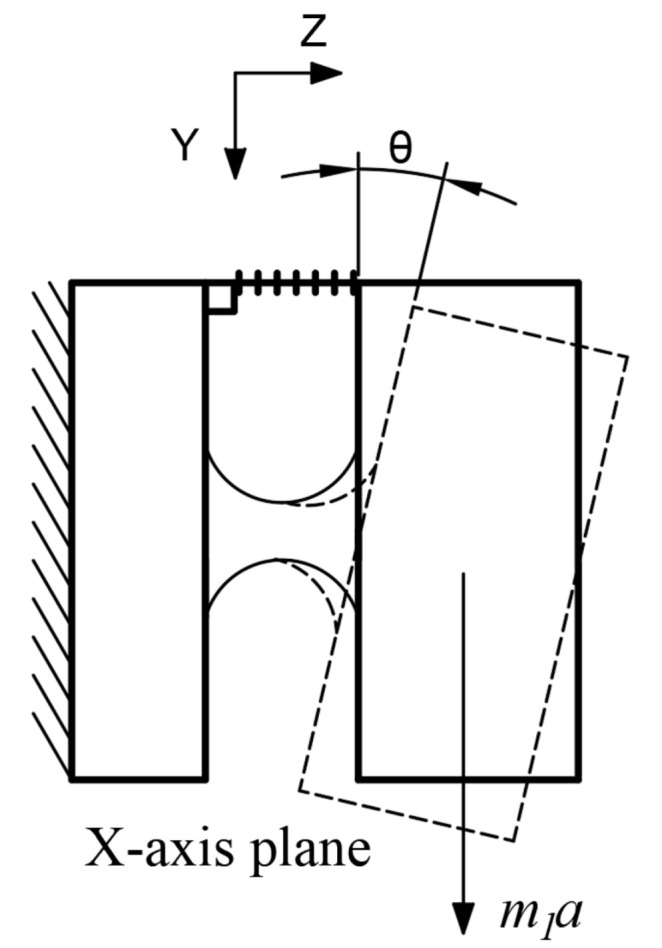
Force analysis diagram of x-direction sensor.

For an acceleration *a* applied in the y-direction of the accelerometer, the moment balance equation gives

(2) $$m_{1}ad_{1}-k\Delta l_{1}h-M_{x}=0$$

where *m*_1_ is the mass of the mass block of the elliptical biaxial hinge sensing structure, *k* is the elastic coefficient of the optical fiber, Δ*L*_1_ is the elongation of a grating of length *L*_1_ under the tension of the mass block, and *M_x_* is the rotational potential energy of the mass block under the action of inertial force:

(3) $$M_{x}=\frac{\theta_{x}}{C_{x}}$$

where *θy* is the deflection angle of the mass around the y-axis under x-axis acceleration, as shown in Figure 3.

When the mass block deflects (rotates), the FBG is stretched. For a very small rotational angle, $\theta_{x}\approx \tan\theta_{x}$, and we obtain

(4) $$\theta_{x}\approx \tan\theta_{x}=\frac{\Delta l_{1}}{h}$$

The elastic coefficient *k* of the fiber is

(5) $$k=\frac{A_{f}E_{f}}{l_{1}}$$

where *A_f_* and *E_f_* are the cross-sectional area and elastic modulus of the fiber, respectively. According to the parameters shown in Figure 3, and assuming that the mass density is uniform, the distance *d*_1_ from the mass center to the center of the elliptical hinge is

(6) $$d_{1}=a+\frac{c}{2}$$

where *a* is the length of the semi-major axis of the elliptical hinge and *c* is the height of the mass along the x-axis. For t/bt≠w/bw, the corner flexibilities *C_x_* and *C_y_* of the elliptical double-axis flexible hinge rotating [28] around the x- and y-axes, respectively, are

(7) $$C_{x}=\left[\begin{array}{c} \frac{6a}{8Eb_{t}^{3}b_{w}c_{1}{\left(c_{1}-1\right)}^{2}{\left(c_{1}-c_{2}\right)}^{2}\left(c_{1}^{3}-c_{1}^{2}c_{2}+2c_{1}^{2}-2c_{1}c_{2}{+c}_{1}-c_{2}\right)\sqrt{\left(c_{1}^{2}-1\right)\left(c_{2}^{2}-1\right)}}\times \\ \;\;\;\;\;\;\;[\left(6c_{1}^{3}c_{2}-4c_{1}^{4}-2c_{1}^{2}c_{2}^{2}+c_{1}^{2}-c_{2}^{2}\right)\sqrt{\left(c_{1}^{2}-1\right)\left(c_{2}^{2}-1\right)}+\\ \left(4c_{1}^{5}c_{2}-8c_{1}^{3}c_{2}+4c_{1}c_{2}\right)B\sqrt{\left(c_{1}^{2}-1\right)}+\left(16c_{1}^{3}c_{2}-6c_{1}^{2}c_{2}^{2}-4c_{1}c_{2}-2c_{1}^{4}-4c_{1}^{6}\right)A\sqrt{\left(c_{2}^{2}-1\right)}] \end{array}\right]$$

(8) $$C_{y}=\left[\begin{array}{c} \frac{6a}{8Eb_{t}b_{w}^{3}c_{2}{\left(c_{2}-1\right)}^{2}{\left(c_{2}-c_{1}\right)}^{2}\left(c_{2}^{3}-c_{1}c_{2}^{2}+2c_{2}^{2}-2c_{1}c_{2}{+c}_{2}-c_{1}\right)\sqrt{\left(c_{1}^{2}-1\right)\left(c_{2}^{2}-1\right)}}\times \\ \;\;\;\;\;\;\;[\left(6c_{1}c_{2}^{3}-4c_{2}^{4}-2c_{1}^{2}c_{2}^{2}+c_{2}^{2}-c_{1}^{2}\right)\sqrt{\left(c_{1}^{2}-1\right)\left(c_{2}^{2}-1\right)}+\\ \left(4c_{1}c_{2}^{5}-8c_{1}c_{2}^{3}+4c_{1}c_{2}\right)A\sqrt{\left(c_{2}^{2}-1\right)}+\left(16c_{1}c_{2}^{3}-6c_{1}^{2}c_{2}^{2}-4c_{1}c_{2}-2c_{2}^{4}-4c_{2}^{6}\right)B^{2}\sqrt{\left(c_{1}^{2}-1\right)}] \end{array}\right]$$

in which

(9) $$\left\{\begin{array}{c} \begin{array}{c} c_{1}=1+t/\left(2b_{t}\right)\\ c_{2}=1+w/\left(2b_{w}\right)\\ A=arctan\left(\frac{c_{1}+1}{\sqrt{c_{1}^{2}-1}}\right) \end{array}\\ B=arctan\left(\frac{c_{2}+1}{\sqrt{c_{2}^{2}-1}}\right) \end{array}\right\}$$

where *t* and *w* denote the minimum thicknesses along the x- and y-axes, respectively, of the double axis hinge, *b_t_* and *b_w_* denote the short half-axis lengths along the hinge x- and y-axes, respectively, and *c*_1_, *c*_2_, *A*, and *B* are the intermediate variables for auxiliary calculation.

The sensitivity of the acceleration sensor is given by the ratio of the shift in wavelength of the FBG to the acceleration magnitude. By applying Equation (2) to Equation (1), the theoretical calculation sensitivity of the acceleration sensor can be expressed as

(10) $$S_{y}=\frac{\Delta \lambda }{a}=\frac{\left(1-P_{e}\right)\lambda_{B}\varepsilon_{f}}{a}=\frac{\left(1-P_{e}\right)\lambda_{B}}{l_{2}}\frac{md_{1}}{kh_{1}+1/h_{1}C_{x}}$$

where *P_e_* is the elastic-optic coefficient, Δ*λ* is the wavelength shift of the FBG, *λ_B_* is the central wavelength of the FBG, *ε_f_* is the strain in the fiber, and Δ*L_1_* is the elongation of the fiber grating under the pulling action of the mass block. A similar sensitivity formula can be obtained for an acceleration of *a* in the y-direction:

(11) $$S_{x}=\frac{\Delta \lambda }{a}=\frac{\left(1-P_{e}\right)\lambda_{B}\varepsilon_{f}}{a}=\frac{\left(1-P_{e}\right)\lambda_{B}}{l_{1}}\frac{md_{1}}{kj/2+2/jC_{y}}$$

When the mass is only affected by acceleration along the y-axis, the general equation of dynamics becomes

(12) $$\frac{d}{t}\left(\frac{\partial L}{\partial {\dot{\theta }}_{x}}\right)-\frac{\partial L}{\partial \theta_{x}}=0$$

where *L* is the kinetic energy of the system. An analysis of the sensor system reveals that

(13) $$L=T_{m}-V_{f}-V_{j}$$

where *T_m_* is the kinetic energy as a function of the angular velocity of the mass:

(14) $$T_{m}=\frac{1}{2}J_{x}{\dot{\theta }}_{x}{}^{2}$$

and *V_f_* and *V_j_* are the energy consumed by the motion of the components of the system, that is, the energy accumulated by the optical fiber and hinge:

(15) $$V_{f}=\frac{1}{2}k{\left(\frac{\theta_{x}h}{2}\right)}^{2}$$

(16) $$V_{j}=\frac{1}{2}\frac{1}{C_{x}}\theta_{x}{}^{2}$$

By substituting Equations (13) to (16) into Equation (12), the dynamic equation of the biaxial hinge system is obtained as

(17) $$J_{x}{\ddot{\theta }}_{x}+(k{\left(h/2\right)}^{2}+1/C_{x})\theta_{x}=0$$

The resonant frequency of the available system is then

(18) $$f_{y}=\frac{1}{2\pi }\sqrt{\frac{k{\left(h/2\right)}^{2}+1/C_{x}}{J_{x}}}$$

and the moment of inertia *J_x_* is

(19) $$J_{x}=m\frac{c^{2}+h_{1}{}^{2}}{12}+md_{1}{}^{2}$$

The natural frequency of acceleration in the x-direction can also be derived as

(20) $$f_{x}=\frac{1}{2\pi }\sqrt{\frac{k{\left(j/2\right)}^{2}+1/C_{y}}{J_{y}}}$$

and the moment of inertia *J_y_* is

(21) $$J_{y}=m\frac{c^{2}+j^{2}}{12}+md_{1}{}^{2}$$

#### 2.1.2. Z-Direction Theoretical Formula

**Figure 4:**
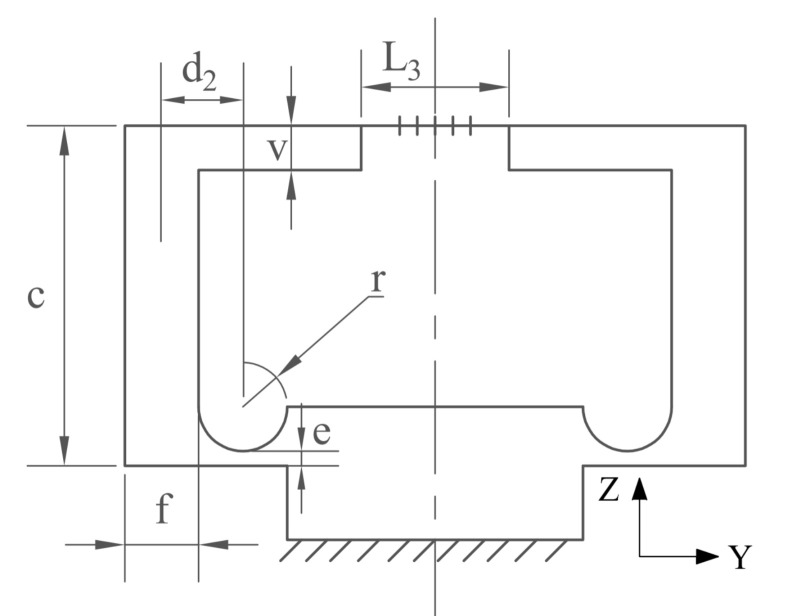
Simplified schematic of z-direction sensor.

A simplified symmetrical hinge structure for measuring the acceleration along the z-direction and its size parameters is shown in Figure 4.

**Figure 5:**
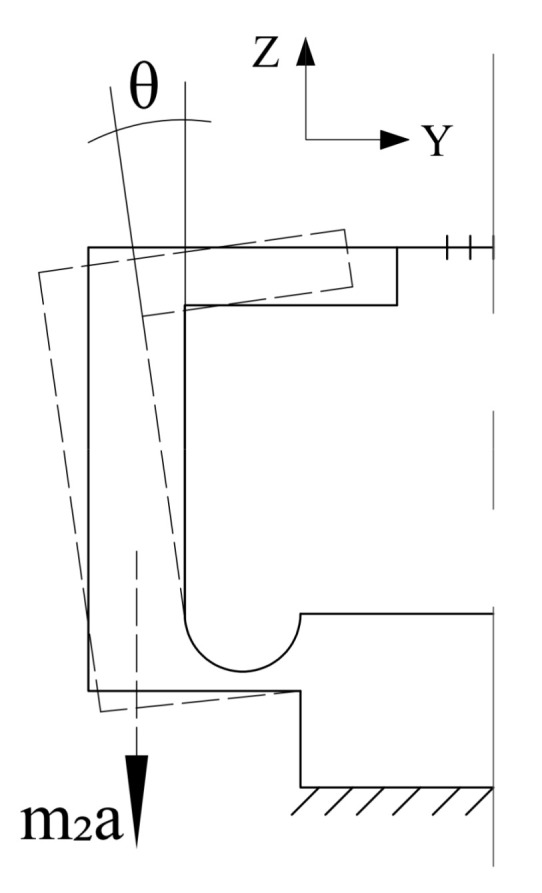
Force analysis diagram of z-direction sensor.

Because the structural dimensions on both sides of the sensor are completely symmetrical, one of the hinges can be analyzed separately. When an acceleration of *a* is applied in the z-direction, the mass rotates around the hinge center, and the motion form is shown in Figure 5. From the moment balance equation, we obtain

(22) $$m_{2}ad_{2}-k\Delta l_{3}c-M_{z}=0$$

where *m*_2_ is the mass of the single-mass block, *d*_2_ is the projection along the y-axis of the distance from the center of mass of the block to the hinge center, Δ*L*_3_ is the variation in length on the z-plane of the suspended optical fiber, and *M_z_* is the rotational potential energy at this time:

(23) $$M_{z}=\frac{\theta_{z}}{C_{z}}$$

where *θ_z_* is the rotational angle of the single-side hinge under the action of acceleration along the z-axis. For $\theta_{Z}=\Delta L_{3}/C_{Z}$, the rotational angle stiffness *C_Z_* of the circular arc single-side hinge [29] around the x-axis is

(24) $$C_{Z}=\frac{12}{Eea^{2}} [\frac{p^{3}\left(3p^{2}+4p+2\right)}{\left(p+1\right){\left(2p+1\right)}^{2}}+\frac{6p^{4}\left(p+1\right)}{{\left(2p+1\right)}^{\frac{5}{2}}}arctan\sqrt{2p+1}]$$

where *E* is the elastic modulus of the hinge material and *p* is the intermediate variable for calculation:

(25) $$p=\frac{r}{e}$$

where *d*_2_ is the projection along the y-axis direction of the distance from the barycenter of mass to the center of the unilateral hinge:

(26) $$d_{2}=r+\frac{\frac{cf^{2}}{2}-\frac{v{\left(n+2r-\frac{l_{3}}{2}\right)}^{2}}{2}}{cf+v\left(n+2r-\frac{l_{3}}{2}\right)}$$

The sensitivity in the actual z-direction is twice that of the sensitivity obtained from the single-side calculation:

(27) $$S_{z}=\frac{\Delta \lambda }{a}=\frac{2\left(1-P_{e}\right)\lambda_{B}\varepsilon_{f}}{a}=\frac{4\left(1-P_{e}\right)\lambda_{B}}{l_{3}}\frac{m_{2}d_{2}}{kc+1/cC_{z}}$$

If the moment of inertia of the mass around the hinge center is *J_z_*, the kinetic energy of the mass will be

(28) $$T_{m}=\frac{1}{2}J_{z}{\dot{\theta }}_{z}{}^{2}$$

At this time, the elastic potential energy of a single-side hinge will be

(29) $$V_{j}=\frac{1}{2C_{z}}\theta_{z}{}^{2}$$

the dynamic equation of the system will then be

(30) $$J_{z}{\ddot{\theta }}_{z}+\left(kc^{2}+1/C_{z}\right)\theta_{z}=0$$

and the resonance frequency in the z- direction can be obtained as

(31) $$f_{z}=\frac{1}{2\pi }\sqrt{\frac{kc^{2}+1/C_{z}}{J_{z}}}$$

### 2.2. Dimensional Parameter Optimization

#### 2.2.1. Z-Axis Dimensional Optimization

Because of its structural characteristics, the z-axis sensing properties of the proposed three-dimensional FBG acceleration sensor change only with the motion of the symmetrical unilateral hinge and mass block structural parameters. In contrast, the unilateral hinge structural parameters affect the sensing characteristics along all three axes. Therefore, in analyzing the influence of sensor size parameters, priority is given to the relevant parameters in the z-direction and the control variable method is primarily used.

The primary parameters affecting the z-direction sensing performance are *r*, *e*, *c*, *f*, *v*, and *n*, with *v* and *n* determining the size of the mass block structure used to control the grating packaging distance. Relative to their impact on the mass block size, the impact of these factors on the sensor can be ignored. To simplify the analysis process, *v* and *n* can be taken as fixed values and their impacts on the sensor disregarded.

**Figure 6:**
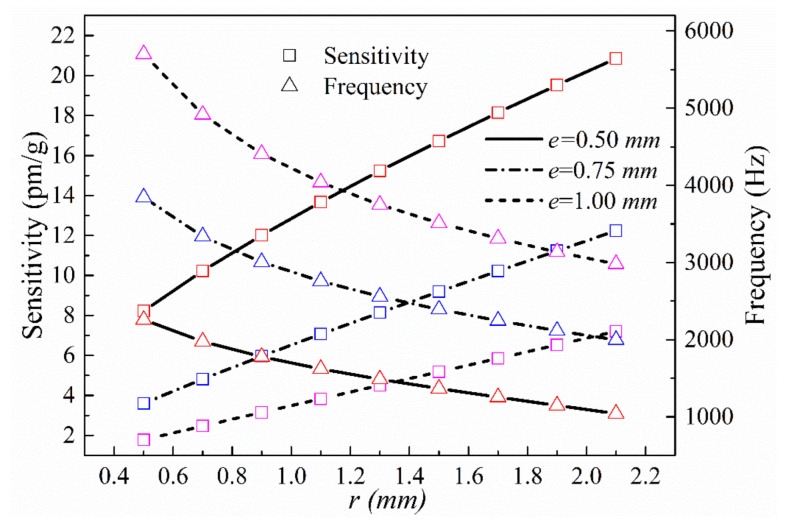
Sensitivities and natural frequencies in the z-direction as a function of *r* for different values of *e*.

The size of the hinge is the most important factor affecting the hinge sensor. The impacts of the single-hinge size parameters, *r* and *e*, on the z-direction sensing performance were analyzed. The other sensor parameters—*c*, *f*, *j*, *n*, and *v*—were set to 10, 3, 20, 5, and 2 mm, respectively. The theoretical sensor z-direction resonance frequency and sensitivity for *e* = 0.5, 0.75, and 1 mm, respectively, over the range 0.5 mm ≤ *r* ≤ 1.5 mm are shown in Figure 6.

**Figure 7:**
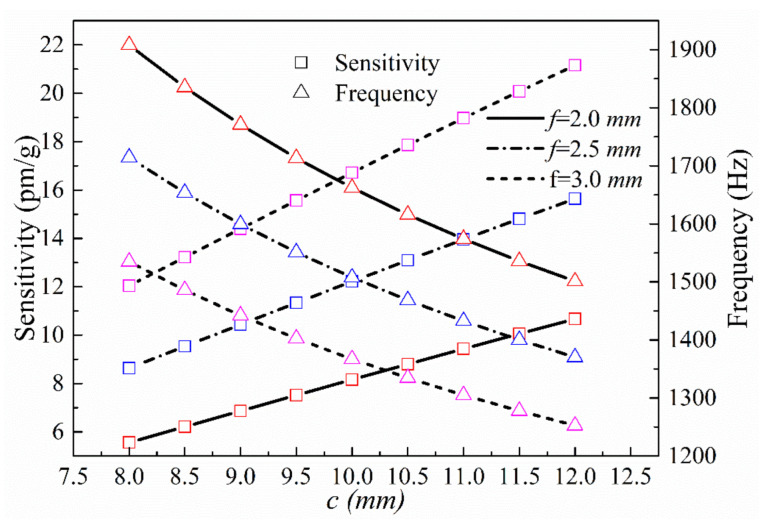
Sensitivities and natural frequencies in the z-direction as a function of *c* at different values of *f*.

We then analyzed the impacts of the z-direction mass block dimensions *c* and *f* on sensing performance. Setting *r*, *e*, *j*, *n*, and *v* as 1.5, 0.5, 20, 5, and 2 mm, respectively, we calculated the theoretical resonance frequencies and sensitivities in the z-direction over the range of 8 mm ≤ *c* ≤ 12 mm for *f* = 2, 2.5, and 3 mm, respectively (Figure 7).

**Figure 8:**
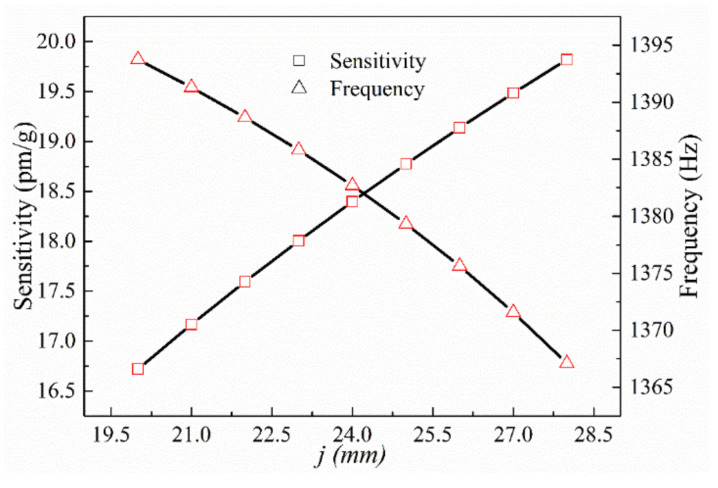
Sensitivity and natural frequency in the z-direction as a function of *j*.

Finally, we analyzed the impacts of mass and hinge thickness, *j*, on the z-direction sensing performance. Setting *r*, *e*, *c*, *f*, *n*, and *v* as 1.5, 0.5, 10, 3, 5, and 2 mm, respectively, we calculated the theoretical resonance frequency and sensitivity in the z-direction over the range of 20 mm ≤ *j* ≤ 28 mm (Figure 8).

It is seen from the calculated results that the z-direction sensitivity of the sensor increases with the radius of the single-side arc hinge, *r*, and decreases with the thickness of the thinnest section of the hinge, *e*. The sensitivity also increases with the mass length and height, *f* and *c*, respectively, and with the overall structure thickness, *j*. The resonance frequency follows trends opposite to those followed by sensitivity. It is also seen that the z-direction results are in line with the relationship between sensitivity and resonance frequency. The primary parameter affecting the z-axis sensitivity of the sensor is the size of the unilateral hinge; decreasing *e* without changing the overall size of the sensor will significantly increase the sensitivity. Although changing the size of the mass block increases the sensitivity, it will also increase the mass and volume of the sensor.

#### 2.2.2. X and Y-Axis Dimensional Optimizations

**Figure 9:**
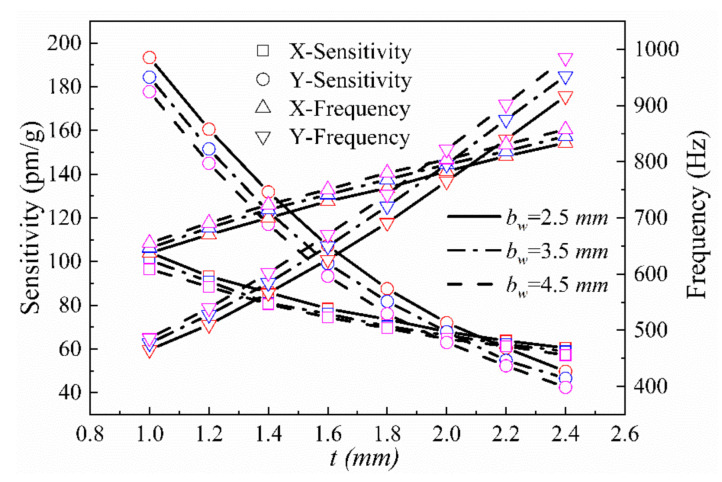
Sensitivities and natural frequencies in the x- and y-directions as a function of *t* at different values of *b_t_*.

**Figure 10:**
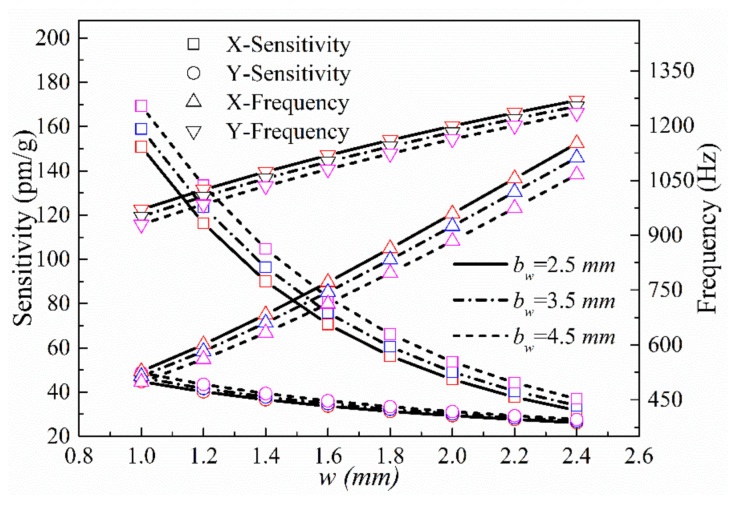
Sensitivities and natural frequencies in the x- and y-directions as a function of *w* at different values of *b_w_*.

**Figure 11:**
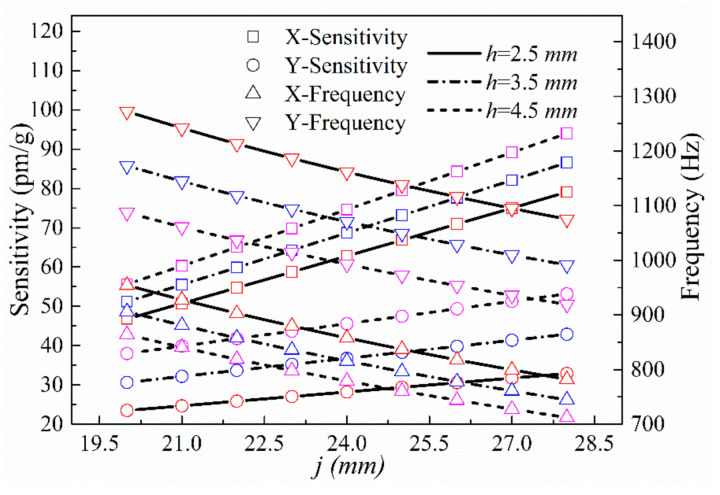
Sensitivities and natural frequencies in the x- and y-directions as a function of *j* at different values of *h*.

**Figure 12:**
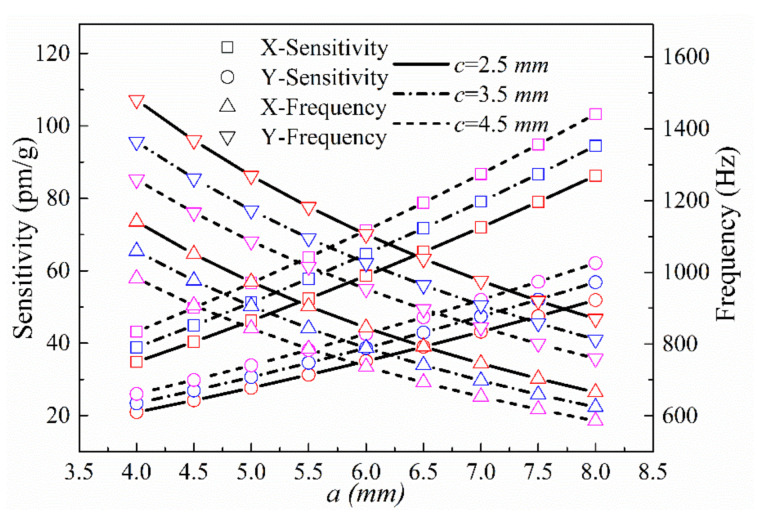
Sensitivities and natural frequencies in the x- and y-directions as a function of *a* at different values of *c*.

The primary structures determining the performance of the sensor along the x- and y-directions are the double axis hinge and the size of the mass block structure above the sensor. The x-axis plane of the double axis hinge primarily determines the sensing performance in measuring acceleration along the y-direction; similarly, the y-axis plane primarily determines the sensing performance along the x-direction. To simplify the analysis process, the structure above the sensor was assumed to be a cuboid mass. The primary parameters affecting the sensing performance in the x- and y-directions are *a*, *t*, *w*, *b_t_, b_w_*, *h*, *j*, and *c*, and the dimensions of the equivalent mass block—*h*, *j*, and *c*—can be controlled by changing the quality parameters along the z-direction. The size of the double axis hinge was optimized by examining the following four relationships:

(1) The impacts of the minimum thickness, *t*, of the hinge on the x-axis plane and the short half-axis length, *b_t_*, of the elliptical notch on the x- and y-axis sensing performance of the double axis hinge were investigated. The parameters *a*, *w*, *bw*, *h*, *j*, and *c* were set to 5, 2, 3, 10.5, 20, and 10 mm, respectively, and *t* was varied over the range 1 mm ≤ *t* ≤ 2.4 mm. The theoretically calculated sensor resonance frequencies and sensitivities in the x- and y-directions at *b_t_* = 2.5, 3.5, and 4.5 mm are shown in Figure 9.

(2) The impacts of the thickness *w* of the thinnest hinge on the y-axis plane and the length *b_w_* of the short half-axis of the elliptical notch on the sensing performance in the x- and y-directions were investigated. The parameters *a*, *t*, *b_t_*, *h*, *j*, and *c* were set to 5, 3, 3, 10.5, 20, and 10 mm, respectively, and *b_w_* was varied over 1 mm ≤ *b_w_* ≤ 2.4 mm. The theoretical resonance frequencies and sensitivities of the sensor in the x- and y-directions at *w* = 2.5, 3.5, and 4.5 mm are shown in Figure 10.

(3) The impacts of the lengths *h* and *j* of the equivalent mass on both planes along the x- and y-directions on sensing performance were investigated. The parameters *a*, *t*, *w*, *b_t_*, *b_w_*, and *c* were set to 5, 3, 2, 3, 3, and 10 mm, respectively, and *j* was varied over the range 20 mm ≤ *j* ≤ 28 mm. The theoretical resonance frequencies and sensitivities of the sensor in the x- and y-directions at *h* = 8.5, 10.5, and 12.5 mm are shown in Figure 11.

(4) The impacts of the same-size parameters of the two-way sensing structure—the long half-axis *a* of the double axis hinge and the height *c* of the equivalent mass—on the sensing performance in the x- and y-directions were analyzed. The parameters *t, w, b_t_, b_w_, h,* and *j* were set to 3, 2, 3, 3, 10.5, and 20 mm, respectively, and *a* was varied over the range 4 mm ≤ *a* ≤ 8 mm. The theoretically calculated resonance frequencies and sensitivities of the sensor in in the x- and y-directions at *c* = 9, 10, and 11 mm are shown in Figure 12.

The above solutions show that the sensitivity of the sensor in the x- and y-directions decreases with the short half-axis radius *b_t_* and the minimum width *t* on the x-axis plane of the elliptical biaxial hinge; however, the effect on the y-axis sensitivity is much greater than that on the x-axis sensitivity. The sensitivity of the sensor in the x- and y-directions decreases with the radius *b_w_* of the short half-axis and the width *w* of the thinnest part of the elliptical biaxial hinge on the y-axis plane; in this case, the effect on the sensitivity along the x-axis is greater. The sensitivities on both the x- and y-axes increase with *j* and *h*, with *j* having more influence on the sensitivity along the x-axis and *h* having more influence on the sensitivity along the y-axis. The sensitivities along the x- and y-axes increase with the length *a* of the long half-axis and the height *c* of the equivalent mass. Comprehensive analysis reveals that the x-axis sensitivity of the sensor is primarily related to the length of the long half-axis of the double-axis hinge and the size of the y-axis plane hinge, whereas the y-axis sensitivity is primarily related to the length of the long half-axis of the double-axis hinge and the size of the x-axis plane hinge. Increasing the size of the uppermost mass block will increase the sensitivities in both directions but will also increase the overall volume of the sensor. Therefore, in designing the sensor, we first selected the dimensions of the biaxial hinge along all directions to obtain appropriate sensitivities along the two axes, and then determined the size of the upper structure.

Based on the above analysis of the influence of the sensor size parameters on its performance, the dimensions of *e*, *r*, *a*, *t*, and *w* were set to 0.5, 1.5, 4, 3, and 2 mm, respectively. The other dimensions were selected according to the degree of difficulty in processing and the mass and volume requirements of the sensor. All dimensional parameters are listed in Table 1. By substituting the selected size, material, and optical fiber parameters into the formulas presented in Section 2, we obtained the following theoretical FBG three-dimensional accelerometer parameters: (1) an x-axis sensitivity and resonance frequency of 60.8 pm/g (approximately) and 879.6 Hz, respectively; (2) a y-axis sensitivity and resonance frequency of 30.9 pm/g and 1166.4 Hz, respectively; and (3) a z-axis sensitivity and resonance frequency of 18.1 pm/g and 1777.1 Hz, respectively.

## 3. Finite Element Simulation

**Figure 13:**
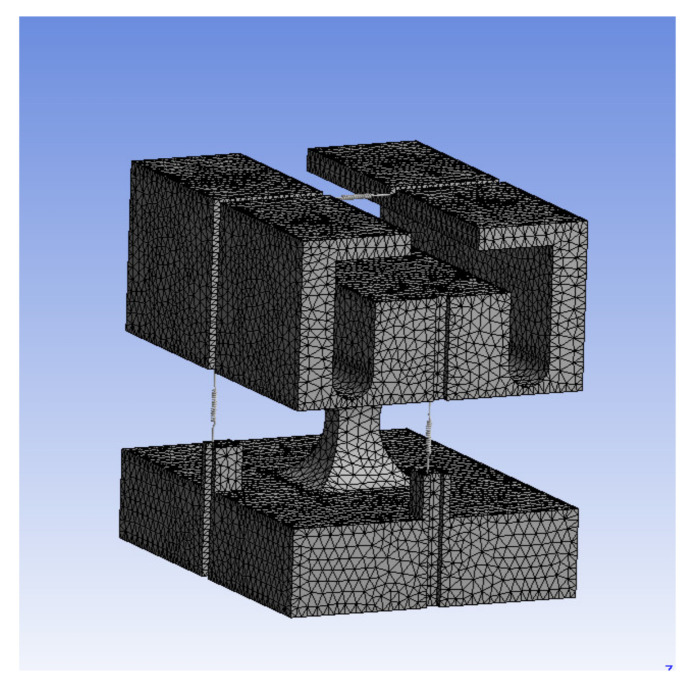
Schematic of accelerometer meshing.

The structural parameters of the three-dimensional accelerometer set according to the actual project requirements and machining capacity are listed in Table 1. Figure 13 shows the design mesh of the three-dimensional accelerometer. For the meshing, a 20-node solid element was selected and 304 steel was used as the material. After imposing a fixed constraint on the bottom of the base, a modal analysis and sensitivity simulation of the sensor were carried out.

### 3.1. Natural Frequency

**Figure 14:**
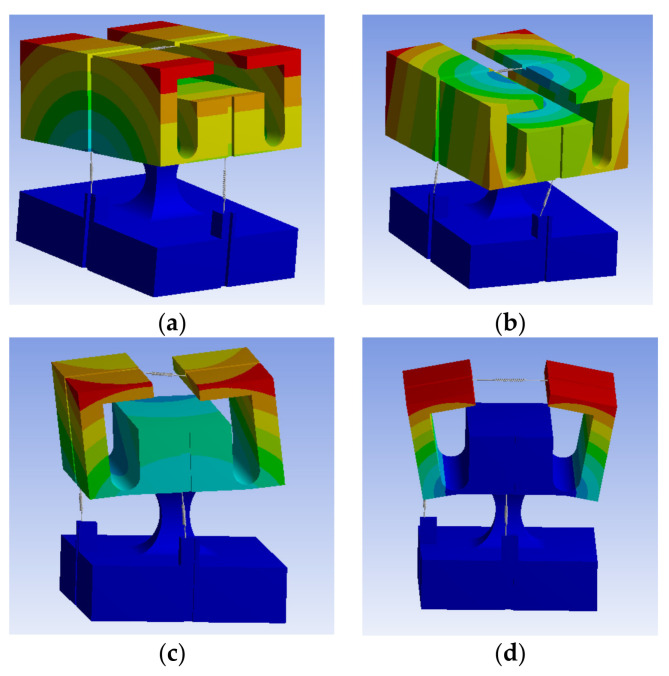
(**a**) First-, (**b**) second-, (**c**) third-, and (**d**) fourth-order modes.

Figure 14a–d shows the first- through fourth-order modal analysis results, respectively, for the three-dimensional accelerometer. The corresponding natural frequencies in the x-, y-, and z-directions are 780.51, 950.17, and 1443.90 Hz, respectively. From the theoretical formulas in Equations (18) and (20), the resonance frequencies are 879.60, 1166.40, and 1777.10 Hz in the x-, y-, and z-directions, respectively, and the errors in the simulation results are 9, 18, and 18%, respectively.

### 3.2. Harmonic Response

**Figure 15:**
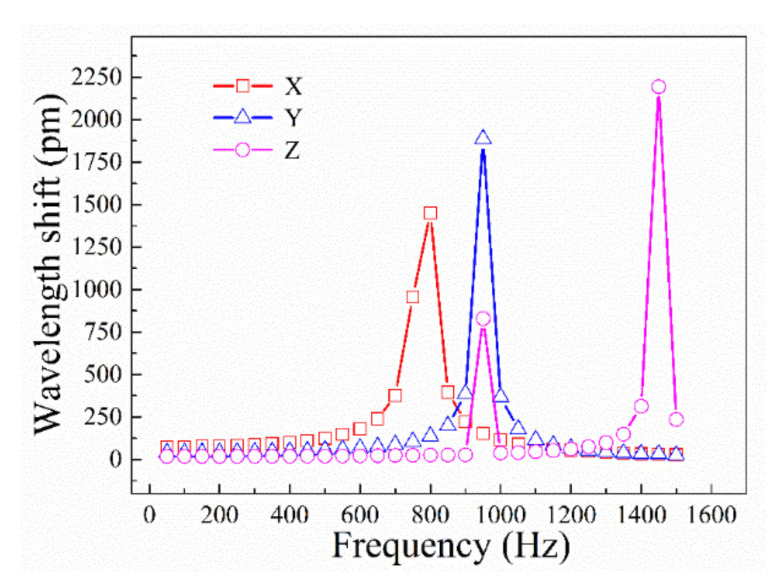
Sensor amplitude frequency curves along the x-, y-, and z-directions.

In the harmonic response analysis, it was assumed that there are no relative displacements between the FBGs and the metal elastic structure. The axial displacements between the two ends of the suspended FBGs and the metal bonding boundary were set equal to the deformation of the FBGs when stretched or contracted. A sine acceleration signal of 1 g was then applied along all three directions of the finite element model of the sensor. To obtain as complete a sensor amplitude frequency curve as possible and ensure that the frequency range of analysis covered the resonance frequency in three directions to the greatest extent possible, the signal frequency applied to the sensor was varied over the range of 0–1500 Hz and the signal amplitude was set as a unit acceleration, i.e., 9.8 m/s^2^. The amplitude frequency curves of the sensor along the three directions are shown in Figure 15.

An analysis of the results in Figure 15 indicates that the amplitude frequency curves of the x-, y-, and z-direction FBGs have peaks at the first-, third-, and fourth-order modal frequencies, respectively. The z-direction amplitude frequency curve has an additional peak at the y-direction resonance frequency, indicating that the sensor units are not independent of each other, but have mutual influence. The motion amplitude of the y-direction resonance is larger than normal, resulting in a center wavelength shift of the z-direction FBG. Figure 15 also shows that the resonance frequencies of the sensor in the x-, y-, and z-directions are approximately 800, 950, and 1450 Hz, respectively. In general, the differences in sensitivity between the sensors in the working frequency band are not more than 10%, indicating that the applicable frequency of the sensor is determined, within a narrow measurable frequency band, by the x-axis frequency.

### 3.3. Sensitivity

**Figure 16:**
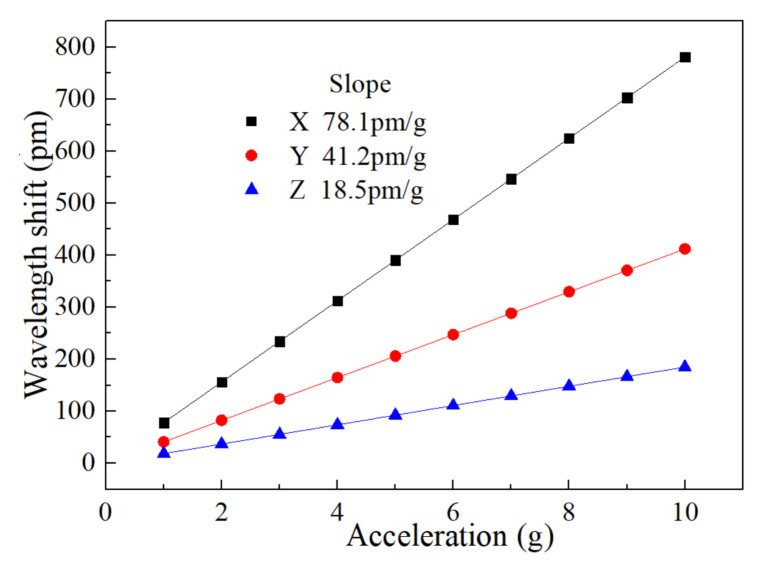
Wavelength shifts in the x-, y-, and z-directions as functions of acceleration amplitude.

We used finite element simulation analysis to calculate the changes in fiber length between the upper and lower fixed points of the three FBGs (Δ*L*_1_, Δ*L*_2_, Δ*L*_3_) corresponding to different acceleration amplitudes. The strain at each FBG was calculated from the change in the fiber length (*ε*_x_ = Δ*L*_1_/*L*_1_, *ε*_y_ = Δ*L*_2_/*L*_2_, *ε*_z_ = Δ*L*_3_/*L*_3_). Using Equation (1), the wavelength shifts of the three FBGs (Δλ_x_, Δλ_y_, Δλ_z_) corresponding to different acceleration amplitudes were then calculated (Figure 16). Based on the slopes of the dotted curves in the figure, the sensitivities of the three-dimensional accelerometer along three main axis directions were obtained as 78.1, 41.2, and 18.5 pm/g, respectively.

## 4. Testing and Discussion

### 4.1. Installation of FBGs

To ensure that the elastic body of the three-dimensional accelerometer moved in all directions with the mass block to properly drive the deformation and stress variation of the FBGs, it was necessary to prestress the gratings during the accelerometer packaging process. Thus, in fabricating a fiber with three FBGs, the spacing between adjacent gratings was designed in advance. In addition, to prevent the gratings from chirping, they were not pasted onto the steel structure.

**Figure 17:**
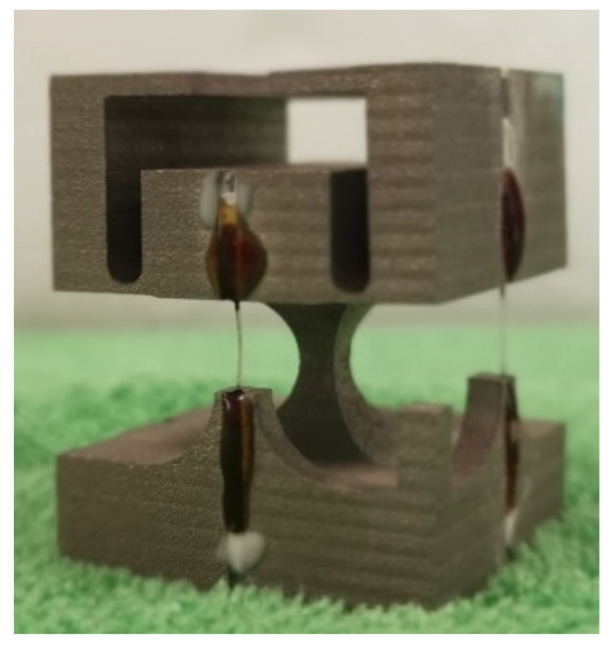
Physical image of three-dimensional acceleration sensor.

After fixing the sensor to a heating table, FBG3 was installed along the y-direction at the top of the core with its fibers placed in the two fiber slots located at the top. With FBG3 continuously prestressed, glue was dripped into the two fiber slots and, following solidification, the prestress loading was eliminated. FBG1 was installed along the z-direction with one end of the grating fixed with adhesive and the other end suspended with a weight to apply stretching. Following packaging and filling with glue, the optical fibers were fixed onto the base. FBG2 was fixed along the z-direction in the same manner as FBG1 along the x-direction. Following the fixing of the three FBGs, the core was fixed into the sensor shell, the protective optical cable sheath was sheathed onto the tail fiber, and the optical cable was fixed onto the shell through the connector. The finished sensor is shown in Figure 17.

### 4.2. Performance Testing

**Figure 18:**
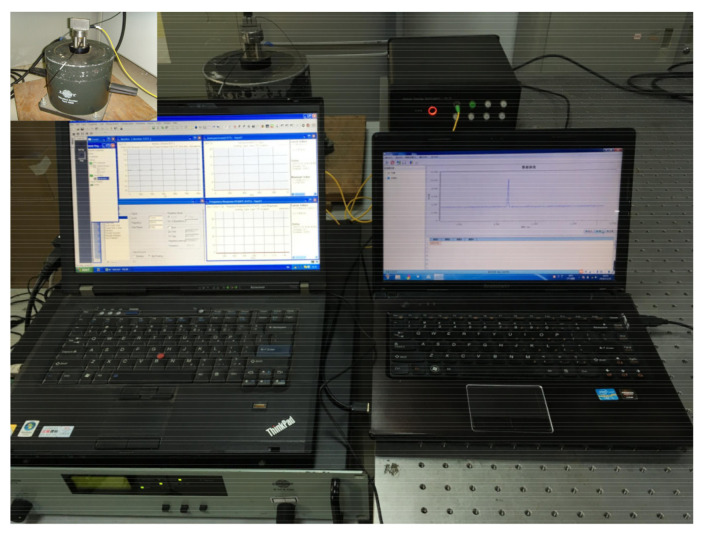
Accelerometer dynamic calibration testing system.

Figure 18 shows the testing system used during the accelerometer dynamic calibration process. The overall experimental system comprised three components:

(1) The vibration sensing system on the left side of the figure comprised a signal amplifier, standard acceleration sensor, modem amplifier, data acquisition front end, and analysis software.

(2) The data acquisition system on the right side of the figure comprised an external OP-FBG2000 acquisition instrument and computer-hosted data acquisition software. The resolution and sampling frequency of the acquisition instrument were 1 pm and 8000 Hz, respectively.

(3) The exciter, located at the upper left corner of the figure, was attached to the accelerometer.

In the experimental tests, the vibration frequency and amplitude were generated and controlled by the shaking table and the changes in the wavelength signal of the FBGs were collected by the acquisition instrument and transmitted to the data acquisition software on the computer. The initial FBG parameters measured by the three-dimensional accelerometer were shown in the Table 2. The experiments were conducted at room temperature (20 °C).

#### 4.2.1. Natural Frequency Testing

**Figure 19:**
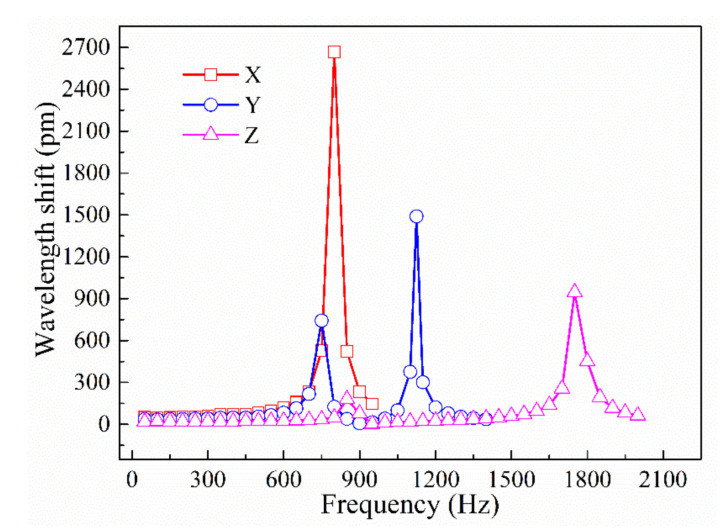
Amplitude frequency curves of accelerometer in x-, y-, and z-directions.

The amplitude frequency curve is an important characteristic index for determining the applicational frequency range of an acceleration sensor. A series of frequency tests were conducted at a fixed acceleration amplitude of 1 g. Figure 19 shows the amplitude frequency curves of the three-dimensional accelerometer along the three principal axis directions. It is seen that the resonance frequencies of the accelerometer in the x-, y-, and z-directions are approximately 800, 1125, and 1750 Hz, respectively, results that are close to those provided by the finite element analysis (780.51, 950.17, and 1443.8 Hz in the respective directions). Prior to reaching the resonance frequency, each amplitude frequency curve has a flat area with a small slope, which is called the measurable range of the accelerometer; in this range, the influence of frequency variation on the sensitivity of the accelerometer can be ignored. The working frequency ranges in the x-, y-, and z-directions are 0–300, 0–500, and 0–1000 Hz, respectively.

#### 4.2.2. Sensitivity Testing

**Figure 20:**
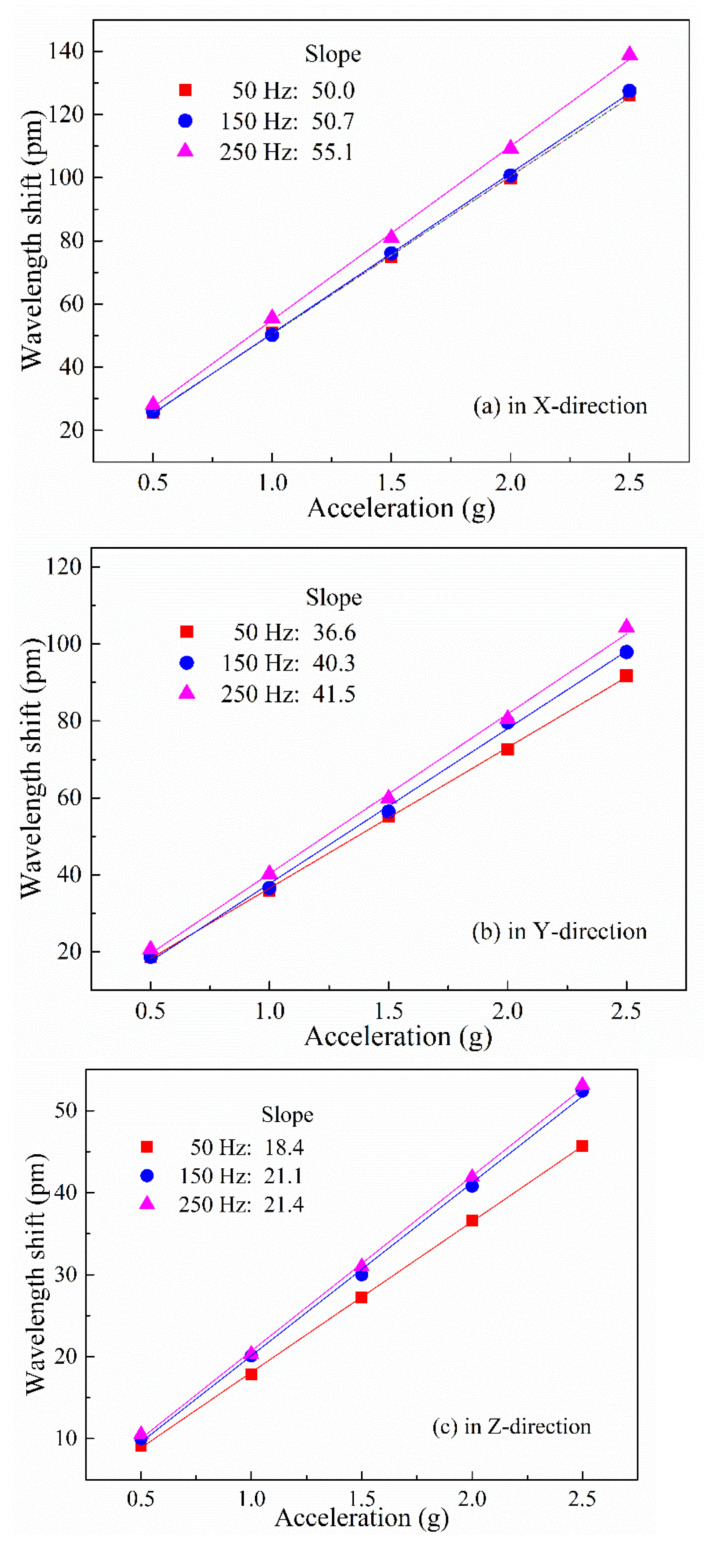
Wavelength shift of accelerometer as a function of acceleration amplitude in the (**a**) x-, (**b**) y-, and (**c**) z-directions.

Sensitivity testing was carried out over an acceleration amplitude range of 0–2.5 g at vibration frequencies of 50, 150, and 250 Hz in each of the three principal axis directions. Figure 20a–c shows the wavelength shifts as functions of acceleration amplitude in the x-, y-, and z-directions of the accelerometer, respectively. It is seen that the dynamic response of the three-dimensional accelerometer has a high degree of linearity, with sensitivities in the x-, y-, and z-directions of 51.9, 39.5, and 20.3 pm/g, respectively, which are close to the respective figures obtained from the finite element analysis.

#### 4.2.3. Anti-Interference Testing

**Figure 21:**
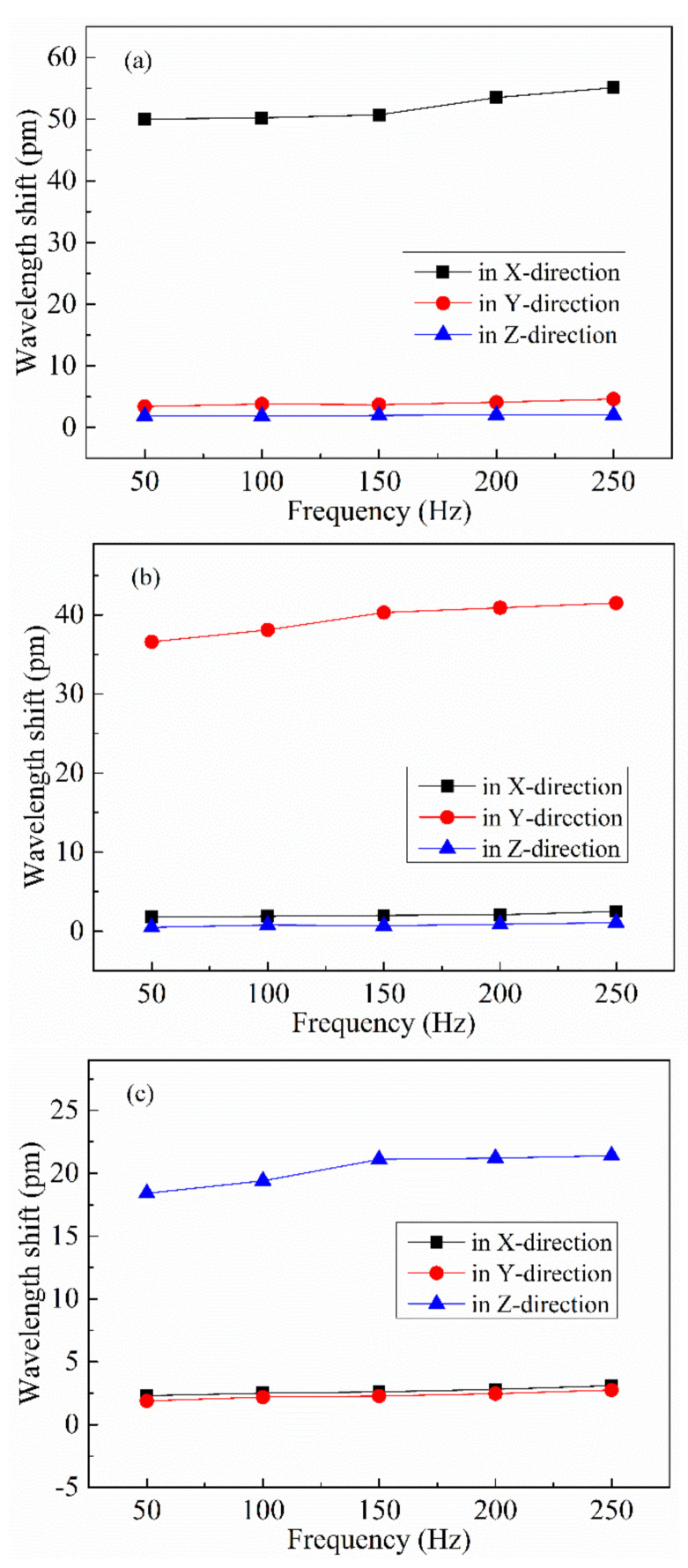
Wavelength shift versus frequency curves of accelerometer in the x-direction (**a**), y-direction (**b**), and z-direction (**c**).

To measure the interdimensional interference within the sensor, 50-Hz, 100-Hz, 150-Hz, 200-Hz, and 250-Hz with 1 g sinusoidal accelerations were applied along all three directions of the sensor and the wavelength changes experienced by each FBG were measured. Figure 21a gives the wavelength shift versus frequency curve of the sensor when the vibration happens along the X-direction in the range of 50–250 Hz; it can be calculated that the sensitivities in the y-direction (Syx = 3.92 pm/g) and the z-direction (Szx = 1.95 pm/g) are 7.55% and 3.76% of the sensitivity along the main axis (Sxx = 51.9 pm/g), respectively. Figure 21b gives the wavelength shift versus frequency curve of the sensor when the vibration happens along the y-direction in the range of 50–250 Hz; it can be calculated that the sensitivities in the x-direction (Sxy = 2.1 pm/g) and z-direction (Szy = 2.4 pm/g) are 5.32% and 6.08% of the sensitivity along the main axis (Syy = 39.5 pm/g), respectively. Figure 21c shows the wavelength shift versus frequency curve of the sensor when the vibration happens along the z-direction in the range of 50–250 Hz; it can be calculated that the sensitivities in the x-direction (Sxz = 1.7 pm/g) and y-direction (S_YZ_ = 0.8 pm/g) are 8.37% and 3.94% of the sensitivity along the main axis (S_ZZ_ = 20.3 pm/g), respectively.

The results of the anti-interference tests indicate that the sensitivity of the secondary axis of the sensor in each direction is less than 10% of the sensitivity of the main axis and, therefore, the three-dimensional accelerometer has a high degree of lateral stability and strong anti-interference ability.

### 4.3. Dynamic Response Testing

**Figure 22:**
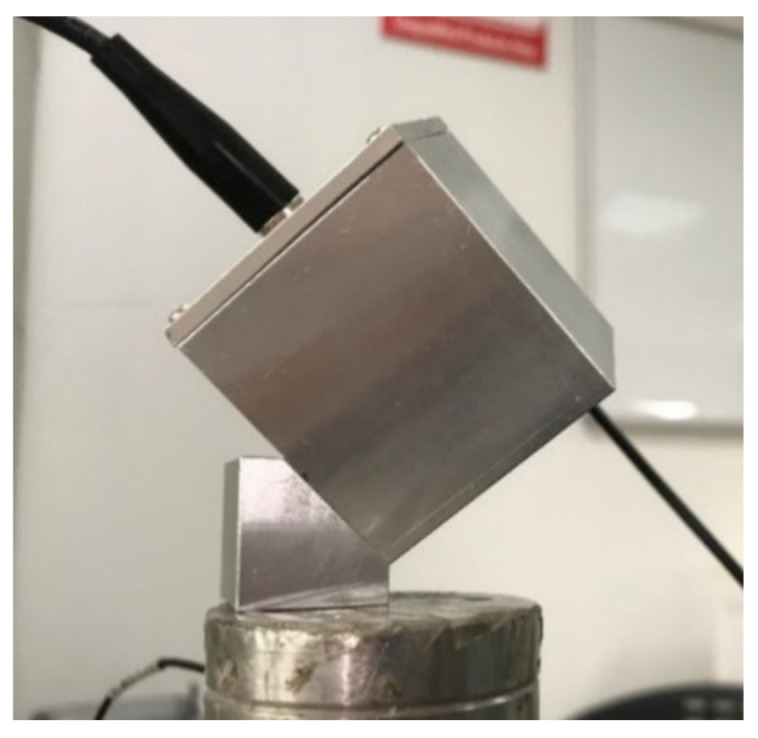
Installation of sensor in dynamic test experiment.

Dynamic response testing in which 2 g, 50 Hz sinusoidal acceleration signals were applied to the sensor at 45 degrees relative to the positive direction of each axis of the sensor were conducted. To carry out the tests, an aluminum transition block with a groove was added between the sensor and the shaking table (Figure 22). The sensor was fixed into the groove of the transition block with adhesive. By fixing the transition block to the shaking table, the direction of motion of the shaking table relative to the three axial directions of the sensor could be maintained at 45°.

**Figure 23:**
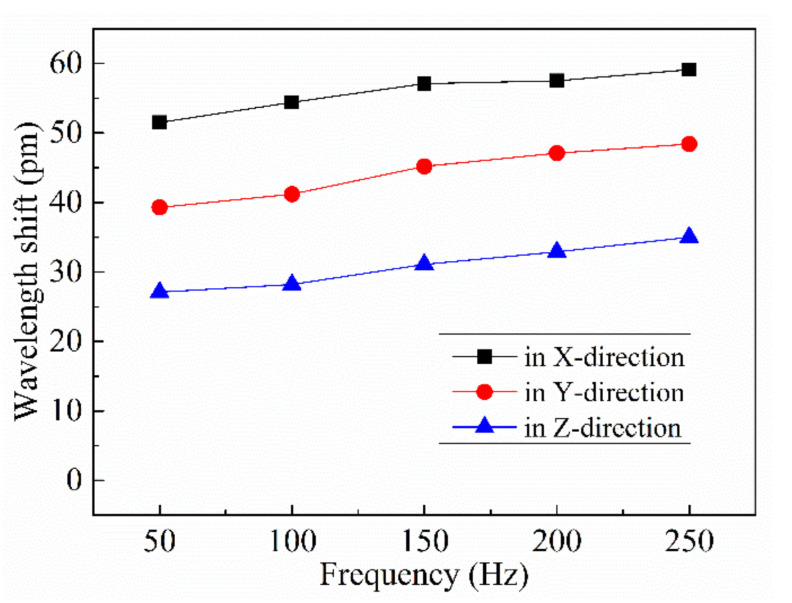
Wavelength shift versus frequency curves of the accelerometer when the vibration happens along a special direction (A_X_ = 1 g, A_Y_ = 1 g, A_Z_ = 1.414 g).

Over a series of experiments, the wavelength shifts of the three FBGs were recorded. The wavelength shift versus frequency curves of the sensor are given in Figure 23, and the average sensitivities in the x-, y-, and z- directions are $\Delta \lambda_{X}=55.92$ pm, $\Delta \lambda_{Y}=44.24$ pm, and $\Delta \lambda_{Z}=30.86$ pm, respectively. The acceleration values corresponding to the sensor sensitivities in the x-, y-, and z-directions were 1.09, 1.12, and 1.52 g, respectively. Following vector addition, this equates to an acceleration measured by the sensor of approximately 2.18 g, with an error of 9.0% relative to the actual excitation signal.

## 5. Conclusions

In this study, we added an additional one-dimensional sensing structure to a two-axis hinge structure to fabricate a three-dimensional FBG accelerometer in which three FBGs are installed within an integrated oscillator structure. The oscillator structure is composed of a single material and comprises a mass block, biaxial elliptical hinge, single-side circular arc hinge, and base. We established a theoretical model of the proposed three-dimensional accelerometer and used finite element software to simulate its dynamic response characteristics with optimal structural parameters. The size of the optimized accelerometer is only 25 mm × 25 mm × 30 mm. Sensing data obtained from numerous laboratory tests revealed that the sensitivities of the proposed three-dimensional accelerometer in in the x-, y-, and z-directions are 52.1, 39.5, and 20.1 pm/g, respectively, with corresponding natural frequencies of 800, 1125, and 1750 Hz, respectively. The proposed three-dimensional accelerometer has the advantages of reduced volume and weight and the ability to carry out simultaneous measurements of acceleration along multiple dimensions.

## Figures and Tables

**Table 1:** Parameters of FBG three-dimensional accelerometer.

Parameter	Numerical Value (mm)
*a*	4
*t*	3
*w*	2
*b_t_*	3
*b_w_*	3
*n*	5
*i*	7
*r*	1.5
*e*	0.5
*c*	11.5
*f*	2.5
*v*	1.5
*j*	26

**Table 2:** Initial parameters of FBGs.

Axial	Parameter	Numerical Value
X	central wavelength	1544.3482 nm
	period	0.1 um
	reflectivity	>70%
	grid length	5 mm
Y	central wavelength	1533.4438 nm
	period	0.1 um
	reflectivity	>70%
	grid length	5 mm
Z	central wavelength	1558.2875 nm
	period	0.1 um
	reflectivity	>70%
	grid length	5 mm

## Data Availability

Not applicable.
